# Skeletal regeneration in the brittle star *Amphiura filiformis*

**DOI:** 10.1186/s12983-016-0149-x

**Published:** 2016-04-22

**Authors:** Anna Czarkwiani, Cinzia Ferrario, David Viktor Dylus, Michela Sugni, Paola Oliveri

**Affiliations:** Department of Genetics, Evolution and Environment, University College London, London, UK; Department of Biosciences, University of Milan, Milan, Italy; Centre for Mathematics, Physics and Engineering in the Life Sciences and Experimental Biology, University College London, London, UK; Present address: Department of Ecology and Evolution & Center for Integrative Genomics, University of Lausanne, Lausanne, Switzerland; Research Department of Genetics, Evolution and Environment, University College London, Room 426, Darwin Building, Gower Street, London, WC1E 6BT UK

**Keywords:** Brittle star, Echinoderms, Skeleton, Regeneration, Proliferation, C-lectin, p58b, p19

## Abstract

**Background:**

Brittle stars regenerate their whole arms post-amputation. *Amphiura filiformis* can now be used for molecular characterization of arm regeneration due to the availability of transcriptomic data. Previous work showed that specific developmental transcription factors known to take part in echinoderm skeletogenesis are expressed during adult arm regeneration in *A. filiformis*; however, the process of skeleton formation remained poorly understood. Here, we present the results of an in-depth microscopic analysis of skeletal morphogenesis during regeneration, using calcein staining, EdU labeling and *in situ* hybridization.

**Results:**

To better compare different samples, we propose a staging system for the early *A. filiformis* arm regeneration stages based on morphological landmarks identifiable in living animals and supported by histological analysis. We show that the calcified spicules forming the endoskeleton first appear very early during regeneration in the dermal layer of regenerates. These spicules then mature into complex skeletal elements of the differentiated arm during late regeneration. The mesenchymal cells in the dermal area express the skeletal marker genes *Afi-c-lectin, Afi-p58b and Afi-p19*; however, EdU labeling shows that these dermal cells do not proliferate.

**Conclusions:**

*A. filiformis* arms regenerate through a consistent set of developmental stages using a distalization-intercalation mode, despite variability in regeneration rate. Skeletal elements form in a mesenchymal cell layer that does not proliferate and thus must be supplied from a different source. Our work provides the basis for future cellular and molecular studies of skeleton regeneration in brittle stars.

**Electronic supplementary material:**

The online version of this article (doi:10.1186/s12983-016-0149-x) contains supplementary material, which is available to authorized users.

## Background

Regeneration, the ability to regrow missing body parts after self-induced or traumatic amputation, has fascinated scientists for a long time. In the context of adult organisms, this developmental process varies extensively among animals (reviewed in [1]) and also among different parts of the same organism. Some animals, such as cnidarians and flatworms, can regenerate the entire body from small fragments [2–5], while others can restore only some cell tissues or particular body parts, as in vertebrates (reviewed in [6]). Echinoderms are marine deuterostomes and constitute an invertebrate phylum containing five extant classes, all possessing extensive regenerative abilities in both adult and larval forms (reviewed in [7]). Animals belonging to this phylum are characterized by 1) penta-radial symmetry, 2) large coelomic cavities, 3) a complex system of fluid-filled canals called the water vascular system and used for various aspects of animal life, 4) a well-developed nervous system, and 5) a calcareous endoskeleton [8]. Understanding how this group of animals regenerate entire body parts formed by different tissue types can provide valuable insight into our understanding of different mechanisms of animal regeneration and their evolutionary origin, and might help to explain why not all animals possess this postembryonic developmental mode.

The most prominent characteristic of all adult echinoderms is the mesoderm-derived skeletal system composed of epidermis-covered ossicles (dermaskeleton) and some internalized skeletal components (e.g.*,* vertebrae of ophiuroids) [8]. The degree of development of the skeleton varies among the different classes, from small isolated ossicles of holothurians to the pervasive skeleton of the sea urchin formed by several different calcified elements such as teeth, body armor and spines [8]. Ossicles have a three-dimensional porous structure formed by crystals of calcite and associated proteins, also referred to as stereom, which provides light but sturdy endoskeletal support [9]. The molecular and cellular mechanisms of skeleton development have been extensively studied in sea urchin embryos [10–12] and they provide the basis for investigations on adult skeleton development, regeneration, and comparison with other classes. In postembryonic skeletogenesis, the formation of spicules and the participation of skeletogenic cells in echinoderm juveniles has been previously described, and major morphological similarities between embryonic and juvenile skeletogenic cells have been observed [13]. Indeed, at both life stages the skeleton is formed by round-shaped mesenchymal cells with filopodia, capable of migrating to the location where new skeleton is deposited [13]. These morphological studies have been complemented by: 1) gene expression analyses, carried out in both sea urchin and starfish, which show that many of the genes involved in sea urchin embryonic skeletogenesis are also expressed in juvenile skeletogenic centers [14, 15]; and 2) proteomic studies that revealed an extended similarity of the molecular make-up of embryonic and adult isolated skeletal elements [16–18]. Adult sea urchin spine regeneration was described over 80 years ago [19]. This process has been studied only in terms of ultrastructure and growth process of the tip of the spine [20, 21]. Recently, a single study investigated the potential role of the Notch signaling pathway in sea urchin regeneration [22], but without providing any insight into the cellular or molecular aspects of this regenerative process. In other echinoderms regeneration is better understood at the level of morphogenesis, including the well-characterized nervous system regeneration in holothurians [23, 24], and arm regeneration in asteroids [25], crinoids [26] and ophiuroids [27]. Nevertheless, the cellular and molecular aspects of specifically skeletal regeneration have not been investigated in detail in those echinoderm clades.

Brittle stars (class: Ophiuroidea) are the most diverse group of echinoderms comprising over 2000 species with a global distribution [28, 29]. Adult brittle stars are able to regenerate their entire arms, making them an appealing system for studying regeneration of adult structures. Brittle star arms are complex structures composed of various tissue types organized in repetitive segments, here referred to as metameric units. Each such unit contains five different skeletal elements-the dermal oral, aboral and lateral shields (or plates), spines, and the internal vertebral ossicles-in addition to a set of two pairs of intervertebral muscles, intervertebral ligaments, and a pair of podia on each side. The radial water canal, the different specialized coelomic cavities (i.e., aboral coelomic canal, neural sinuses) and a radial nerve cord and peripheral sensory neurons are also present throughout the arm segments [30]. *Amphiura filiformis (Afi)*, a burrowing brittle star from the North Sea, has been used to understand various aspects of regeneration in this class [27, 31–34]. Some studies have shown significant variability in regeneration rates of these animals. This is likely due to differences in animal size, traumatic versus self-amputation, length of the lost arm, or the most pertinent function required at the time (i.e., the differentiation of sensory structures or the growth of arm for locomotion and feeding) [31]. This variability highlights the plasticity of the regenerative process of these animals. On the other hand, histological studies, which best describe the early repair and regeneration phases in two ophiuroid species [27], emphasize the consistency of the regenerative process in this class of echinoderms. Recent molecular studies identified genes expressed during regeneration [33, 34]. Importantly, it has been shown that several mesodermal genes including *alx1,* a transcription factor known to play a key role in the sea urchin and sea cucumber skeletogenic gene regulatory networks [10, 35], are expressed during both embryonic skeletogenesis [36] and arm regeneration in *A. filiformis* [37]. Importantly, members of the Cart/Alx3/Alx4 group of transcription factors are also involved in skeletal development in vertebrates [38–40].

Despite these studies, little is known about the cellular process underlying skeletogenesis in adult *A. filiformis*, from the initial appearance of simple mineralized elements (spicules) to the formation of complex skeletal ossicles. Here, we address this lack of basic knowledge by using a combination of approaches to characterize skeletal development at the cellular and molecular level in the regenerating arm of *A. filiformis*. To address the problem of variability, we first divide the regenerative process into five stages using clear morphological landmarks, as done in other regeneration models like salamanders [41] and sea cucumbers [23]; and embryonic developmental models such as chicken embryos [42]. We thus use our new staging system to complete the histological survey presented in [27]. We then employ light microscopy and calcein staining to observe at what stage the primordial skeleton forms and in which domain of the regenerating arm. Additionally, we use histological staining and confocal imaging to further understand the position and morphology of the potential skeletogenic cells. We then identify the cells expressing known skeletogenic marker genes *Afi-c-lectin, Afi-p58b and Afi-p19* using in situ hybridization. Finally, we apply 5-ethynyl-2’-deoxyuridine (EdU) labeling to study cell proliferation in the early and late regenerating arms and to determine if differentiated skeletogenic cells are proliferative. Our work will provide the basis for understanding skeleton regeneration in the brittle star on a cellular and molecular level.

## Results

### Staging of early phases of regeneration based on morphological features

**Fig. 1:**
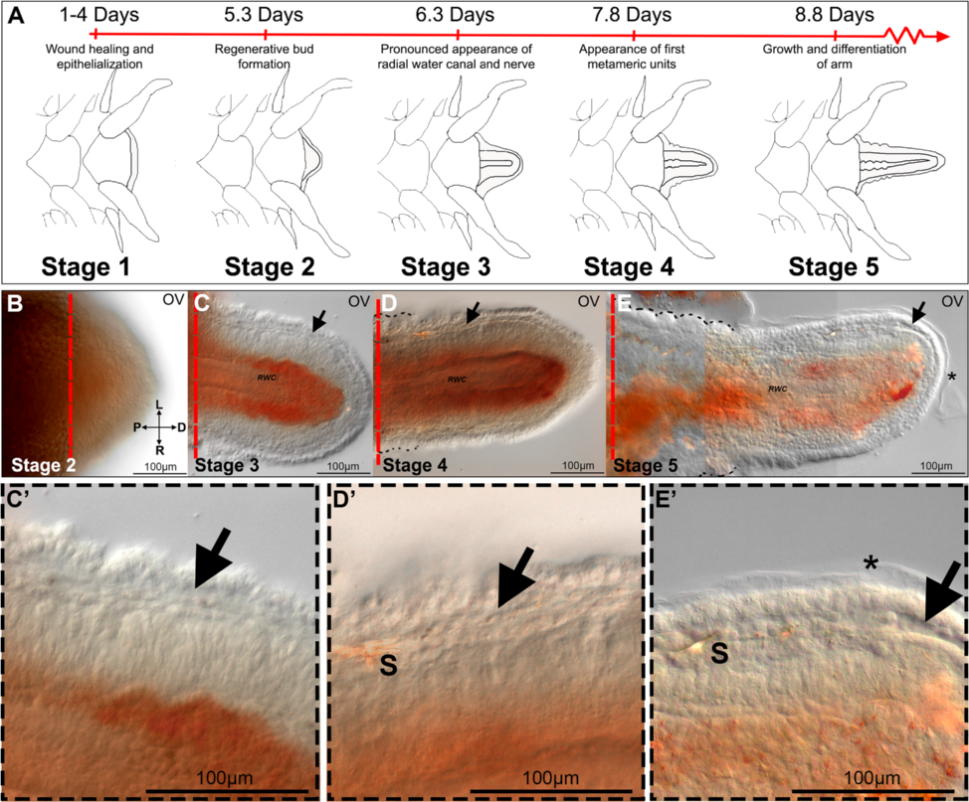
Early stages of arm regeneration in the brittle star *Amphiura filiformis*. A) Schematic diagram of early arm regeneration stages and average timing to those stages, based on morphological landmarks. Stage 1-wound healing and re-epithelialization. Stage 2-regenerative bud formation. Stage 3-appearance of the radial water canal, coelomic cavities (aboral coelomic canal, ectoneural and hyponeural sinuses) and radial nerve in the regenerative bud. Stage 4-appearance of first metameric units (arm segments). Stage 5-advanced extension of arm, formation of several metameric units at proximal end. B–E) Oral view of fixed arms at stages 2 (B), 3 (C), 4 (D) and 5 (E) shown using differential interference contrast (DIC) microscopy. C’) Detail of C focusing on dermal layer. D’) Detail of D. E’) Detail of E. Arrows - the dermal layer, asterisk - cuticle, D - distal, L - left, OV - oral view, P - proximal, R - right, RWC - radial water canal, S - spicule

**Fig. 2:**
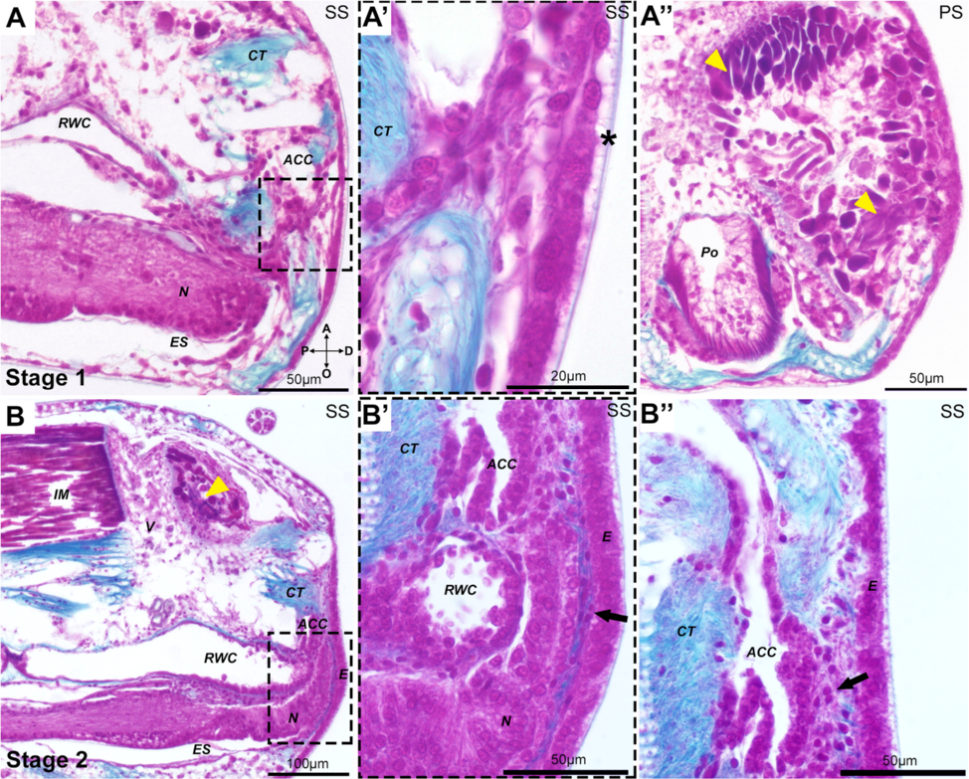
Histological sections of the earliest stages of arm regeneration in the brittle star *Amphiura filiformis*. Sagittal (A and A’) and parasagittal (A”) paraffin sections at stage 1 and sagittal paraffin sections at stage 2 (B) stained with Milligan’s trichrome technique. Collagen stained cyan, all cells labeled pink/magenta. A) The wound is completely healed and re-epithelialization occurs. A’) Detail on the new thin epithelium with a recognizable cuticle (asterisk). A”) Histolysis (arrowheads) of intervertebral muscle bundles proximal to the amputation plane. B) The radial nerve, the radial water canal and the coelomic cavities start regenerating beneath the new epidermis. B’) Detail of the mesenchymal cells (arrow). B”) Detail of the mesenchymal cells at the level of the aboral dermal layer (arrow). A - aboral, ACC - aboral coelomic cavity, CT - connective tissue, D - distal, E - epidermis, ES - ectoneural sinus, intervertebral muscles - IM, N - radial nerve, O - oral, P - proximal, Po - podia, PS - parasagittal section, RWC - radial water canal, SS - sagittal section, V - vertebra

**Fig. 3:**
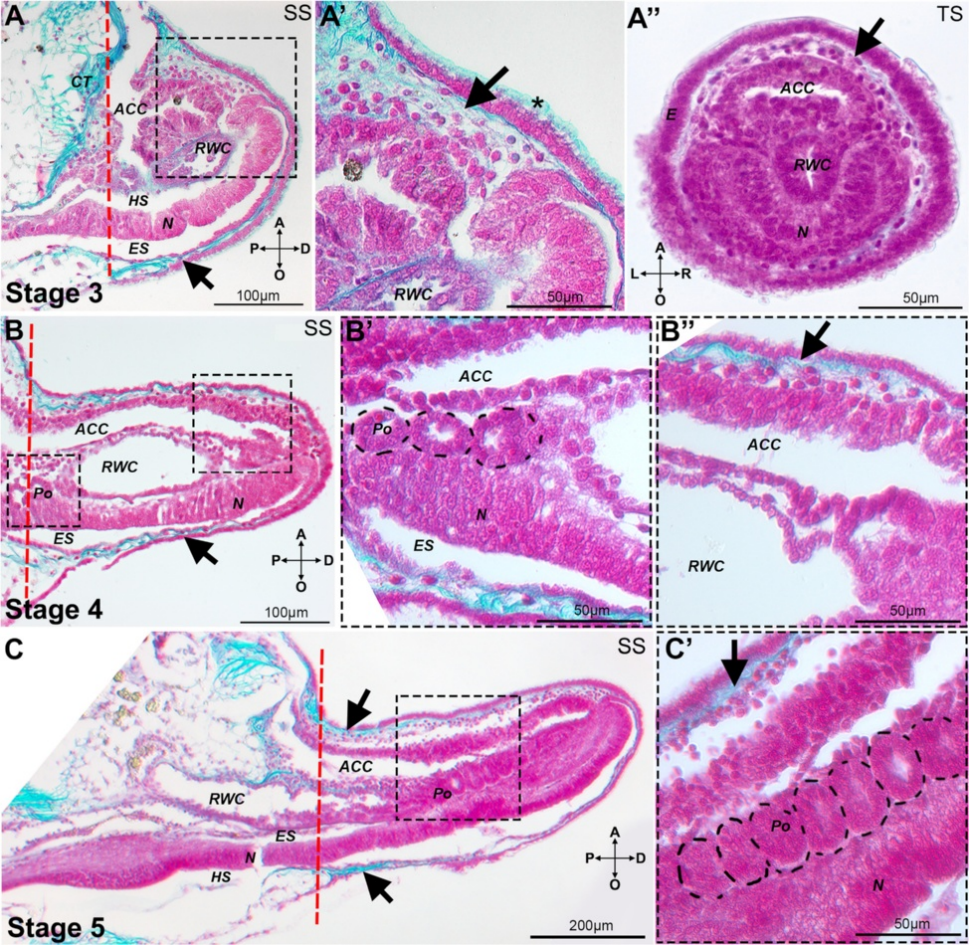
Histological sections of regenerating arms at stages 3, 4 and 5. Sagittal and transverse paraffin sections at stage 3 (A and A”), and sagittal sections of stages 4 (B), and 5 (C) stained with Milligan’s trichrome technique. Collagen stained cyan, all cells labeled pink/magenta. Red dashed line indicates amputation plane. A) The three regenerating axial structures are enveloped by the dermal layer (arrow) and the new epidermis. A') Detail of A showing mesenchymal cells at the level of the aboral dermal layer (arrow) covered by the new epidermis with its cuticle (asterisk). A”) Transverse section of a stage 3 regenerate showing the dermal layer (arrow), developing radial nerve, radial water canal and aboral coelomic cavity. B) The stage 4 regenerate is longer and the radial water canal shows the first signs of podia regeneration. B') Detail of B showing the developing podia (dotted line). B'') Detail of B showing the scattered mesenchymal cells in the aboral dermal layer (arrow). C) Further development and differentiation of the three axial structures at stage 5. C') Detail of developing podia (dotted line). A - aboral, ACC - aboral coelomic cavity, CT - connective tissue, D - distal, E - epidermis, ES - ectoneural sinus, HS - hyponeural sinus, L - left, N - radial nerve, O - oral, P - proximal, Po - developing podia, RWC - radial water canal, R - right, SS - sagittal section, TS - transverse section

The development of a regenerating structure is a dynamic process that proceeds in a step-wise fashion to reconstitute a fully functional structure. To facilitate experimental investigation of arm regeneration in *A. filiformis* we identified five stages, which rely on observable morphological changes. Based on observations of >100 individuals (three of which are shown in Additional file 1: Figure S1) we propose a refined staging system (Fig. 1), which is relevant to the phases of regeneration when cell specification, differentiation and initial arm patterning begin to occur. The presence of clear morphological landmarks is supported by histological analysis carried out on paraffin sections stained with Milligan’s trichrome technique [43], which detects collagen (cyan) and individual cells (pink/magenta) (Figs. 2 and 3). The average duration and standard deviation (S.D.) of time it takes for an arm to regenerate up to a given morphological stage is reported in Fig. 1A, as well as Additional file 1: Figure S1A and B. The timing of regeneration shows a certain degree of variation in agreement to what has previously been reported [31].

All of the stages and corresponding average timings have been compiled based on arms amputated 1cm from the central disc. We categorize stage 1 of regeneration as wound healing and re-epithelialization. This phase occurs between 1 and 4 days post-amputation (dpa) (Fig. 1A; Additional file 1: Figure S1) and involves changes which mediate the closure of the wound and re-epithelialization and remodeling of the existing tissue; however, from whole mount DIC (differential-interference contrast) observations little or no changes are evident at the amputation plane. On the contrary, at this stage histological sections show (Fig. 2) the aboral coelomic cavity (ACC), the ectoneural sinus (ES) and the radial water canal (RWC) are sealed off and the wound is completely re-epithelialized by epidermal cells, already covered by a thin and faint cuticle indicated by the asterisk in Fig. 2A’. The intervertebral muscles adjacent to the amputation site acquire a disorganized pattern and show morphological signs of histolysis: myocytes lose their elongated shape (Fig. 2A”, B). The duration of time between amputation and stage 2 shows the highest variability (Additional file 1: Figure S1B). To assess whether the type of traumatic amputation might have an effect on the rate of the wound healing stage and the appearance of a regenerative bud, we amputated two arms from the same animal: one clean, blunt amputation similar to natural autotomy, and the other skewed (amputation at an angle; Additional file 1: Figure S1C, D). We used 5 animals of the same size to minimize the individual variability and we documented the process of regeneration of each arm for 10 days. These data reported in Additional file 1: Figure S1E reveal a general inter-individual variability in time to reach stage 2, which is not related to the type of amputation.

Once re-epithelialization is accomplished, a small regenerative bud protrudes from the distal end of the stump, in the oral region (Fig. 1A, B; Additional file 1: Figure S1A). We characterize this as stage 2, which occurs on average after 5.3 dpa ±0.91 S.D. (Additional file 1: Figure S1A and B). From whole mount DIC observations the regenerate appears optically homogeneous (Fig. 1B), however histological sections indicate already a certain degree of organization, in which the first outgrowths of the regenerating radial nerve, radial water canal and the aboral coelomic canal are visible (Fig. 2B). A thin layer of connective tissue is present below the wound epidermis where mesenchymal cells are embedded (Fig. 2B’, B”).

Stage 3, which occurs on average 6.3 dpa ±0.48 S.D. (Fig. 1A; Additional file 1: Figure S1A and B), differs from stage 2 in that the regenerate acquires a more organized and complex inner architecture with loss of the external optical homogeneity; at this stage the radial water canal and the sub-epidermal mesenchymal cells become clearly visible in whole mount DIC images (Fig. 1C, C’). Histological sections show the regenerate mainly contains projections of the radial nerve, radial water canal and aboral coelom, which protrude from the amputation plane (Fig. 3A, A”). Mesenchymal cells embedded in collagenous tissues can be detected throughout the dermal layer (Fig. 3A’, A”).

We define stage 4 (on average 7.8 dpa ±0.63 S.D., Fig. 1A; Additional file 1: Figure S1A and B) by the appearance of the first metameric unit, which is formed at the most proximal region of the developing regenerate. A small bulging of the epidermis reveals the early segregation of the regenerating segments (dotted lines, Fig. 1D). Outpocketing of the radial water canal system, which will eventually form the podia, can start to be distinguished in histological sections (Fig. 3B, B’).

Finally, stage 5 occurs on average after 8.8 dpa ±0.63 S.D. (Fig. 1A; Additional file 1: Figure S1A and B). This stage is characterized by several small repetitive units, which can be observed bulging at the proximal side (dotted line, Fig. 1E) with several podia precursors beginning to form along the new regenerate (Fig. 3C, C’).

As the regeneration process progresses metameric units form following a proximal-distal gradient. The late regeneration stages that we use in this work corresponds to the 50 % (2–3 weeks post-amputation) or 95 % (4–5 weeks post-amputation) regeneration stages described before [31, 37]. These arms have a 50 or 95 % differentiation index (DI), which is the ratio between the length of the arm that contains differentiated structures (like spines and podia) and the total regenerate length. The duration of regeneration time is highly variable between stage 5 and the 50 % stage depending on many aspects including length of arm lost, functional requirements, animal size or environmental factors [31].

### Skeletogenesis during early and late regeneration stages

**Fig. 4:**
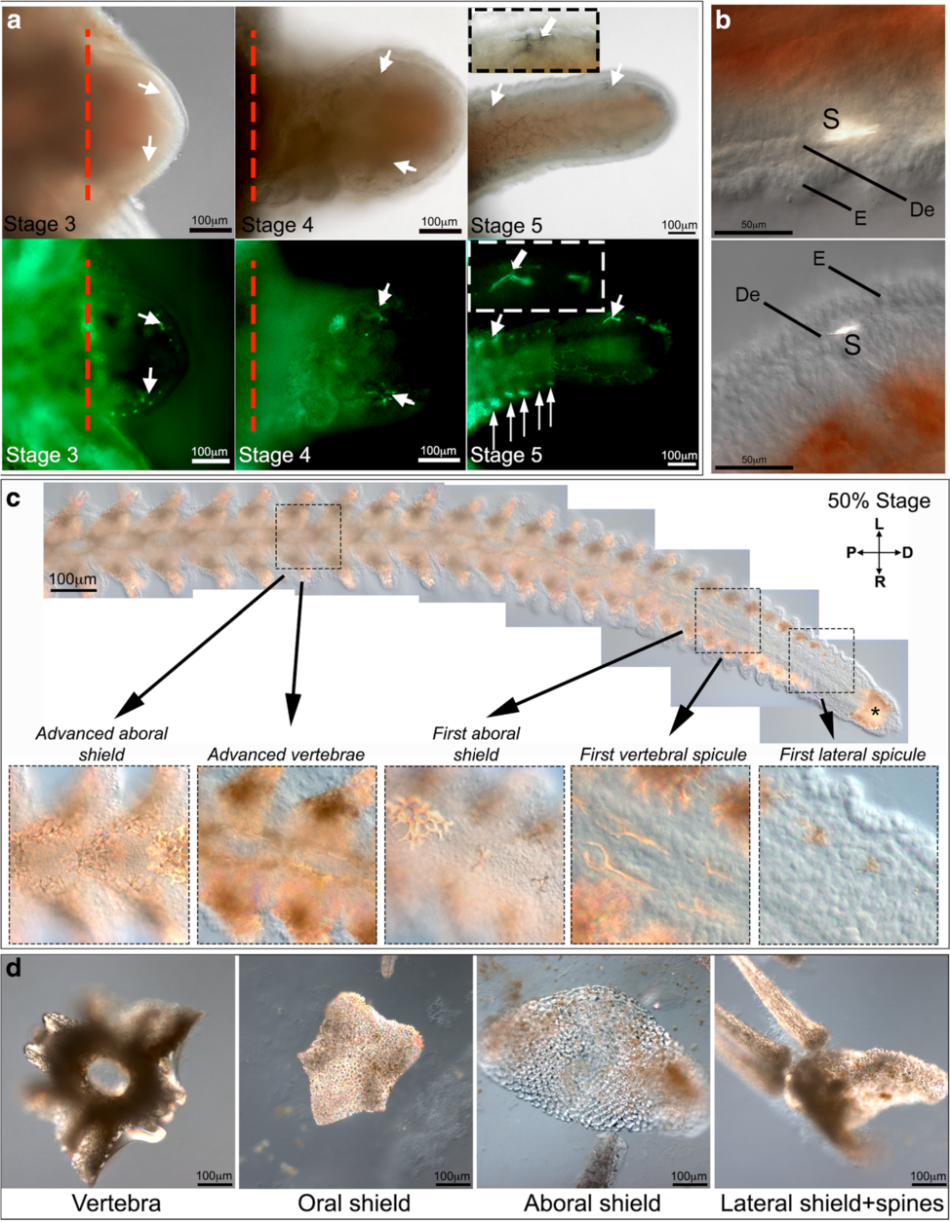
Skeletogenesis during early and late regeneration stages. **a**) DIC (top panels) and fluorescent images (bottom panels) of calcein-labeled spicules at stages 3, 4 and 5. White arrows show individual spicules, which can be compared in both images. Insets show detail of single spicules. **b**) High magnification DIC images showing single spicules localized in the dermal layer. **c**) 50 % differentiated arm showing the formation of the skeletal elements (reflective structures). The highly calcified distal cap (terminal ossicle) forms at the distal-most end of the regenerating arm. The spicules, which will form the lateral shields and spines, appear in the first metameric unit (see inset for detail). Proximally, eight metameric units later, first vertebral spicules can be observed (see inset for detail). At the proximal end of the regenerate skeletogenesis is already very advanced and forms the individual stereomic skeletal elements including the oral, aboral and lateral shields, spines and vertebrae (see insets for detail). **d**) Differentiated skeletal elements in an adult non-regenerating arm. Arrows - calcein-labeled spicules, asterisk - terminal ossicle, E - epidermis, De - dermis. L - left, R - right, P - proximal, D - distal. Red dashed line - amputation plane

To define when and where skeletogenesis occurs during early stages of regeneration in the brittle star arm we combined light transmission microscopy observations of whole regenerates with calcein staining to detect the newly forming mineralized skeleton. Calcein is commonly used to visualize calcium carbonate deposition, which is shown as green fluorescence [44]. Single spots of green fluorescence, corresponding to the forming skeletal primordia, are consistently first observed at stage 3 indicating the earliest stage at which differentiated skeletogenic cells are present (Fig. 4a, *n* = 10). No fluorescence can be detected at stages 1 and 2 (Additional file 1: Figure S2). At stage 4 more elaborate tri-radiated or tetra-radiated skeletal elements called spicules can be observed in the regenerate (Fig. 4a, *n* = 10). At stage 5, the spicules present in the distal end of the regenerate have no obvious pattern of distribution. In contrast, skeletal elements in the newly forming metameric units, at the proximal end of the regenerate, are formed with a typical bilateral distribution that corresponds to where the future lateral shields will be (Fig. 4a , *n* = 10). High magnification of the regenerates at different stages reveals that the single spicules form just below the well-developed epidermis in the dermal layer (Fig. 4b), corresponding to the position of the mesenchymal cells embedded in the collagenous matrix observed in histological sections (Figs. 2 and 3).

Later during regeneration, when the arm reaches the 50 % regeneration stage (50 % DI), the whole maturation of developing skeleton can be observed (Fig. 4c) simply by analysis of subsequent metameric units from proximal (most mature skeleton) to distal (new elements) and using light microscopy. This is due to the birefringent properties of calcified structures. At the distalmost end the distal cap becomes highly calcified (terminal ossicle). The first observable distal metameric unit already contains a tiny tri-radiated lateral spicule (Fig. 4c, first lateral spicule inset). Several metameric units (eight in Fig. 4c) separate the appearance of the first lateral spicules from the appearance of the first vertebral spicules, forming more proximally in the inner layers of the regenerating arm. Contrary to skeletal elements developing during early regeneration and at lateral positions during late regeneration, which always form multi-branched spicules, the vertebrae first appear as two long, non-radiated skeletal rods (Fig. 4c, first vertebral spicule inset). Later, as the skeletal elements mature, they also begin to branch out and the two parallel vertebral elements fuse together at the midline between distal and proximal ends of the metameric unit (Fig. 4c, advanced vertebrae inset). Oral and aboral shields begin to form approximately at the same level as the developing vertebrae (Fig. 4c, first aboral shield inset). In a distal to proximal gradient the segments contain increasingly complex spicule structures that will eventually form the stereom of skeletal ossicles, as is typical for echinoderms (Fig. 4c, advanced shield formation inset). The fully formed structures found in an adult non-regenerating arm are shown in Fig. 4d. A schematic diagram of the organization of individual skeletal shields in the adult non-regenerating arm is shown in Additional file 1: Figure S3.

### Expression of the skeletogenic marker genes in the early and late stages of regeneration

**Fig. 5:**
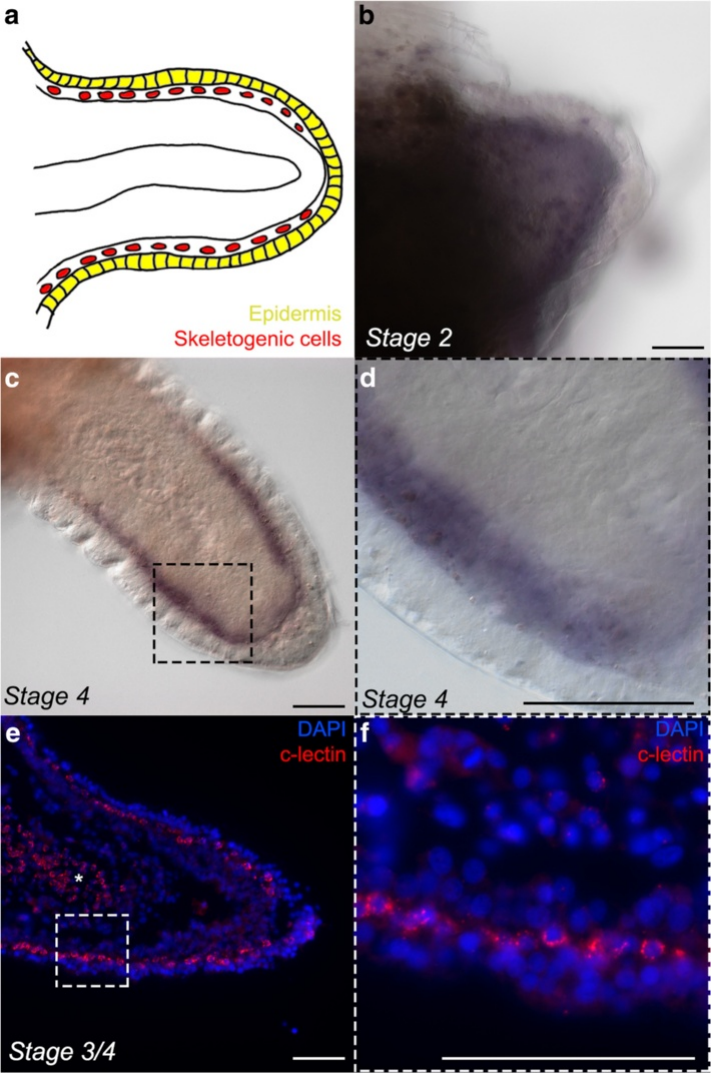
*Afi-c-lectin* expression during early arm regeneration stages. **a**) Schematic representation of a 3/4 stage regenerate. **b**–**d**) Chromogenic whole mount *in situ* hybridization (WMISH) showing expression of gene at stages 2 and 4. **b**) *Afi-c-lectin* is expressed at stage 2 in a broad, sub-epidermal domain. **c**) At stage 4 the expression of *Afi-c-lectin* becomes much more restricted to the sub-epidermal domain. **d**) Higher magnification of arm in **c**. **e**, **f** Fluorescent *in situ* hybridization of sections through stage 3/4 arm counterstained with the nuclear marker DAPI. **e**) Frontal paraffin section showing fluorescent ISH of *Afi-c-lectin* counterstained with DAPI. *Afi-c-lectin* is clearly localized to single cells just beneath the epidermis. Asterisk marks cells in sub-epidermal layer seen from oral view, due to the slanted plane of sectioning along the oral-aboral axis. **f**) Higher magnification of arm in **e**. Scale bars - 50μm

To better characterize the cells involved in skeleton formation during regeneration in the brittle star we assessed the spatial expression pattern of the skeletogenic cell differentiation genes. We first analyzed the closest matching gene, identified in an *A. filiformis* embryonic transcriptome, to the sea urchin C-lectin. This spicule matrix protein containing a C-type lectin domain is expressed specifically in the skeletogenic mesodermal cells [45]. Proteomic studies identified the C-lectin protein as an integral part of biomineralized structures both in sea urchin larval spicules and adult test plates and spines [16, 17], and in ophiuroid adult arm skeletal elements of the species *Ophiocoma wendtii* [46]. The expression of the *A. filiformis c-lectin* gene (Additional file 1: Table S1) has been first examined in the larval stages with extended skeletal elements (i.e., ophiopluteus). Indeed, only a group of mesenchymal cells arranged in a pattern coinciding with the skeletal elements express *Afi-c-lectin* (Additional file 1: Figure S4). We have therefore used it as a marker for skeletogenic cells in adult regenerating arms (Fig. 5). It is first detected by *in situ *hybridization (ISH) at stage 2 of early regeneration in a broad sub-epidermal domain (Fig. 5b). It then becomes much more restricted to the dermal layer only by stage 4 (Fig. 5c, d). This is consistent with the developmental changes that occur between these two stages, when the regenerate acquires a more heterogeneous structure containing morphologically differentiated tissue types. Strong colorimetric staining has the tendency to diffuse in the tissue, rendering the precise boundary of expression not clearly distinguishable. To discriminate whether the expression of *Afi-c-lectin* is restricted specifically to the sub-epidermal mesenchymal cell layer, where spicules form, or extends to other domains of the regenerate, we additionally performed a fluorescent ISH on regenerating arm sections. We found that the expression of this gene is indeed specifically localized to a single cell layer just underneath the epidermis (Fig. 5e, f).

**Fig. 6:**
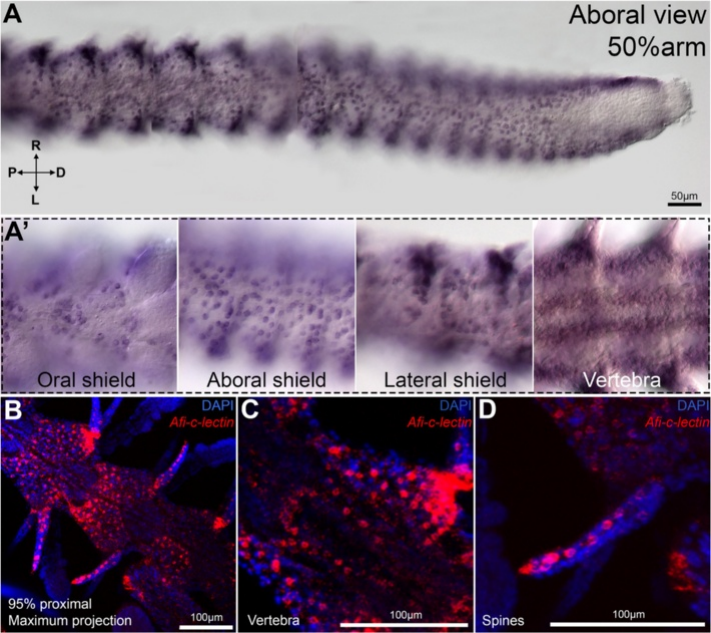
*Afi-c-lectin* expression at 50 and 95 % differentiation stages. A) Chromogenic WMISH of the whole 50 % regenerating arm from proximal differentiated segments to distal undifferentiated segments and terminal cap. At distal-most end staining is localized to sub-epidermal cells. In differentiating metameric units the expression expands to scattered mesenchymal cells covering the areas of future formation of oral, aboral and lateral shields as well as spines. A’) Detail of gene expression in oral shields, aboral shields, lateral shields and vertebrae. B-D) Confocal images of fluorescent WMISH of *Afi-c-lectin* (red) in proximal segments of 95 % differentiated arms counterstained with nuclear stain DAPI (blue). B) Maximum projection showing *Afi-c-lectin* expression in three proximal segments of the arm is localized to cells in the aboral shields and spines. C) Single z-plane projection through one segment in B showing detail of *Afi-c-lectin* expression corresponding to the shape of the growing vertebra. D) Maximum projection through one segment in B showing detail of *Afi-c-lectin* expression along the spine. R - right, L - left, P - proximal, D - distal

Next, we examined the expression of *Afi-c-lectin* in the 50 % DI stage of the regenerating arm (Fig. 6). In the distalmost-undifferentiated segments of the regenerate, *Afi-c-lectin* is expressed in the dermal layer similar to the early stages of regeneration (Fig. 6A). Towards the proximal side of the regenerating arm, its expression becomes more complex, corresponding to the formation and patterning of the different skeletal elements (Fig. 6A). The scattered mesenchymal cells expressing the marker gene are arranged in regular and repetitive patterns, which mirror the pattern of the stereom structure of the oral, aboral, lateral shields with spines and vertebrae (Fig. 6A’) in each of the differentiating segments of the arm. The expression pattern of *Afi-c-lectin* thus coincides specifically with the location and time of appearance of the different skeletal elements; therefore, confirming it as a reliable marker of skeletogenesis during regeneration as well as embryonic development. This is further supported by the expression pattern of *Afi-c-lectin* in the most advanced proximal segments of the 95 % DI regenerating arms (Fig. 6B–D). Two additional genes, identified in a recent embryonic study of *A. filiformis* larval skeleton development [36], were examined to confirm the molecular signature of the mesenchymal cells in the dermis and the cells in the differentiating skeletal elements of late stages of regeneration (Additional file 1: Figure S5). Both *Afi-p19* and *Afi-p58b* have a similar expression pattern to *Afi-c-lectin* at the early stages of regeneration and at the distal end of the 50 % DI stage arms (expression in the dermis, Additional file 1: Figure S5). The genes are more restrictively localized to individual skeletal elements in differentiating proximal segments of the arm (Additional file 1: Figure S5). *Afi-p58b* is expressed in spines and vertebrae, whereas *Afi-p19* is localized to the oral and lateral shields, and vertebrae.

### Differentiated skeletogenic cells are not proliferative

**Fig. 7:**
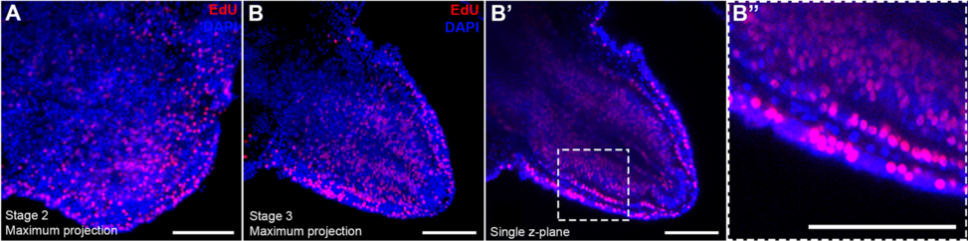
Confocal images showing EdU labeling (red) of early stage regenerating arms counterstained with nuclear stain DAPI (blue). A) Stage 2 maximum projection of confocal z-stack showing widespread cell proliferation in regenerative bud. B) Maximum projection of confocal z-stack of stage 3 arm showing continuing cell proliferation in early regenerate. B’) Single z-plane of B. B”) Detail of B’ showing lack of EdU-labeled cells in dermal layer. Scale bars - 100μm

To test when active cell proliferation is initiated post amputation and if mesenchymal cells - located in the dermal layer where the skeleton is formed - have the ability to proliferate during regeneration, we used the EdU assay to label cells in, or having gone through S-phase during the early stages of regeneration. In normal non-regenerating arms some proliferating cells, labeled by EdU, are identifiable in different tissue types including the epidermis, podia, radial water canal and in cells surrounding the vertebrae (Additional file 1: Figures S6 and S7; *n* = 3). On the contrary, no cells are labeled during the first hours (8–24) post-amputation (Additional file 1: Figure S7; *n* = 3) in the plane of injury. Only at the end of stage 1 (between two and three dpa) is a marked increase in EdU labeling visible at the wound site prior to bulging of the regenerative bud, mainly in correspondence with the position of the radial nerve cord and the radial water canal (Additional file 1: Figure S7; *n* = 3) in the oral half of the metameric unit. Stage 2, which marks the appearance of the regenerative bud, shows extensive cell proliferation in both the epidermis and the inner tissues, containing the outgrowths of the above-mentioned structures (Fig. 7A; *n* = 3).

Five individual arms were used for EdU labeling of stage 3 regenerates. Confocal maximum projections of whole z-stacks also show a large amount of proliferating cells at this stage (Fig. 7B). Notably, closer inspection of individual z-planes reveals that the dermal layer contains the only cells, that are not labeled with EdU, implying that they did not proliferate during the time course of labeling (Fig. 7B’, B”).

**Fig. 8:**
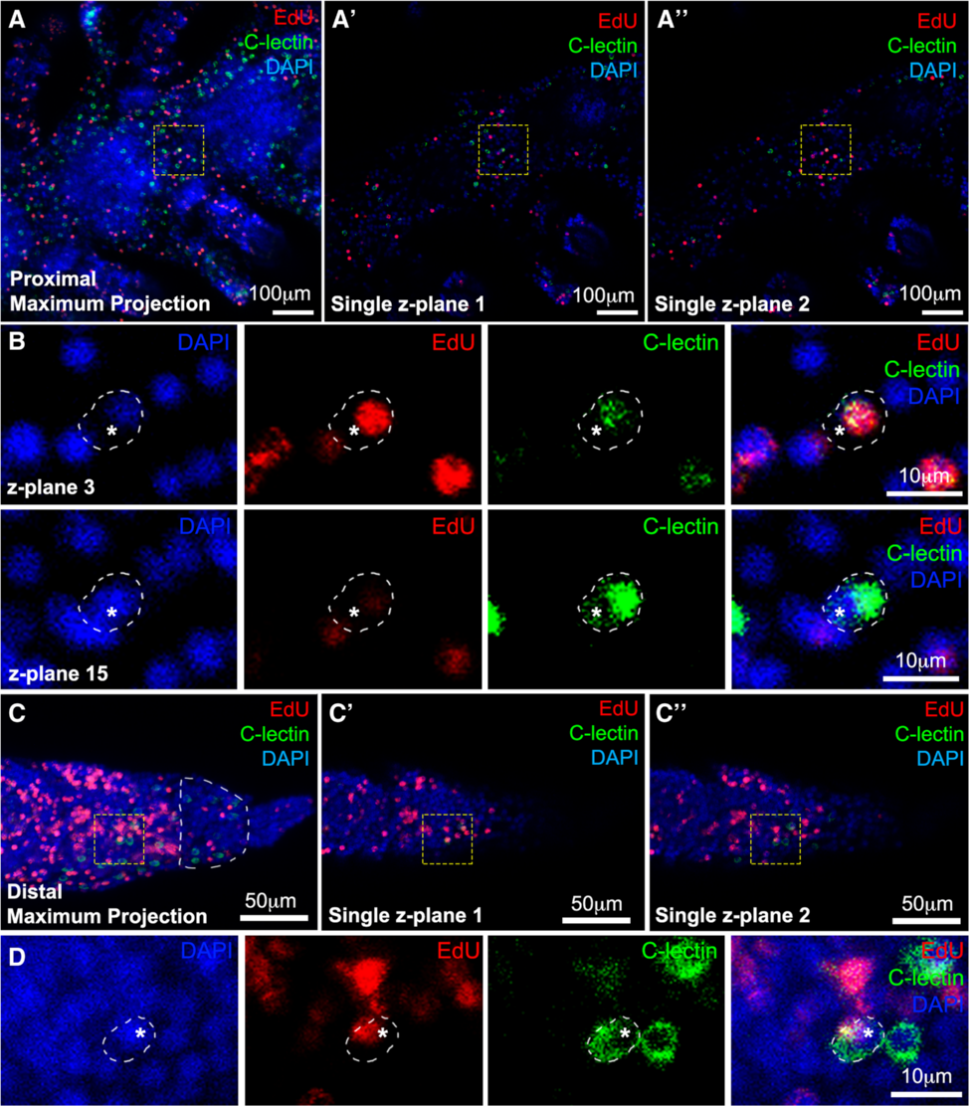
*Afi-c-lectin* expression (green) combined with EdU labeling (red) and counterstained with nuclear stain DAPI (blue) showing that skeletogenic cells do not proliferate. A) Maximum projection of proximal segments of 50 % regenerating arm. A’ and A”) Single z-planes of A showing *Afi-c-lectin* expressing cells do not overlap with EdU-labeled cells. Yellow dashed box shows one case of yellow signal suggesting potential overlap of red and green signals. B) Magnified image in yellow dashed box from A showing cells in z-plane number 3 and z-plane number 15. The EdU labeled red nucleus is seen clearly in z-plane 3 where *Afi-c-lectin* expression is very faint, whereas on z-plane 15 where the whole cytoplasm of the green-labeled *Afi-c-lectin* cell is clearly visible, the nucleus of this cell is labeled with DAPI but not EdU (asterisk). C) Maximum projection of distal-most end of 50 % regenerating arm. White dashed half-circle indicates position of terminal ossicle. C’ and C”) Single z-planes of C showing *Afi-c-lectin* expressing cells do not overlap with EdU-labeled cells. Yellow dashed box shows one case of yellow signal suggesting potential overlap of red and green signals. D) Magnified image in yellow dashed box from C showing cells in single z-plane. Again the nucleus of the *Afi-c-lectin* expressing cell is labeled with DAPI (asterisk) and is in close proximity to the EdU-labeled nucleus causing the yellow overlap signal

As the number of cells that express *Afi-c-lectin* clearly increases during the regeneration process, we investigated whether the cells have the ability to divide at these late stages. To answer this question we performed a fluorescent ISH using *Afi-c-lectin* on arms previously labeled with EdU (*n* = 2). If skeletogenic cells marked by the expression of *Afi-c-lectin* were proliferating, cells with a red nucleus (EdU DNA incorporation) and surrounding green cytoplasm (*Afi-c-lectin* RNA expression) would be observed. Extensive cell proliferation can be observed throughout the regenerating arm at this stage (Fig. 8A). However, closer inspection of individual confocal z-planes shows that none of the green-labeled *Afi-c-lectin* cells overlap with the red EdU^+^ cells in a manner indicating that skeletogenic cells have nuclei in the S-phase of mitosis (Fig. 8; Additional file 1: Table S2; Additional files 2 and 3). Specifically, none of the EdU labeled red nuclei are surrounded by green *Afi-c-lectin* labeled cytoplasm (Fig. 8B, D). This trend is also apparent in the whole 50 % stage arm as shown in the proximal segments (Fig. 8A, B) and the distalmost tip (Fig. 8C, D). Therefore, the *Afi*-*c-lectin* expressing cells do not proliferate throughout the whole arm regeneration process, in agreement with the role of *c-lectin* as a final differentiation gene in skeletogenesis. Notably, the distalmost part (terminal ossicle and podium) (Fig. 8C) of the regenerate contains very few proliferating cells. However, the area just proximal to it is a domain of major accumulation of EdU^+^ cells. This is consistent with previous observations [37], and the expression patterns of genes here reported, and thus we conclude that the regenerating arm proliferative growth zone is located just underneath (proximally) to the terminal ossicle. New metameric units are then added proximal to the growing tip of the regenerate.

## Discussion

In this study we describe the major developmental events occurring during early phases of arm regeneration in the brittle star *Amphiura filiformis*, with a focus on the formation of the calcitic skeleton. The high regenerative capacity of this species, likely evolved to escape predation [31], is ideal for the investigation of the developmental process underlying the regrowth of a completely functional arm constituted by many different cell types and tissues.

The regeneration process, like embryonic development, is characterized by transient stages. At the base of any molecular and cellular investigation of dynamic developmental processes lays the clear understanding of the sequence of events taking place during these stages. Thus, in this study, we first identify five major early regenerative stages (Fig. 1) easily recognizable by external morphology in living animals. These stages subdivide the regenerate starting at the wound healing and repair phase (stage 1), to initial growth (stage 2; also described as blastema in other studies [27, 31]), into more and more complex cellular layers and structures (during stages 3 to 5) that foresee the organization of the future rebuilt arm. There are two previously published staging systems for arm regeneration in *Amphiura filiformis.* The division described by Dupont and Thorndyke [31] only distinguishes an early, uncharacterized phase (called blastema), from two rather advanced stages of regeneration (50 and 95 % DI), in which several metameric units of the regenerate are already present and contain differentiated structures. This staging system, although useful for studying late stages of regeneration and quantifying growth rate, completely bypasses early developmental and morphological events. According to this classification, all our early stages would be classified generally as 0 % DI; therefore, this system is not relevant for our aim of understanding cell specification and morphogenesis. On the other hand, the staging system devised by Biressi et al [27] is focused largely on the very early phases post amputation. This system first subdivides early regeneration into a repair phase (immediately after amputation, 1 dpa) and an early regenerative phase (1–3 dpa), both of which correspond to our stage 1. Our EdU labeling experiments (Additional file 1: Figure S7 B and C) show that the events taking place during this stage do not involve cell proliferation. The two remaining phases described, namely an intermediate regenerative phase (3–12 dpa) and an advanced regenerative phase (11–24 dpa), do not discriminate at high enough resolution the events taking place within this time frame. This is essential to understanding how morphology is generated, and how specification and differentiation of skeletogenic cells occur. For instance, we show that our stage 3 regenerate (average 6.3 dpa) is already characterized by a clear organization of different tissues (Fig. 3A), but is still quite different from stage 4 (average 7.8 dpa) when the metameric units that will form the arm begin to appear. Our revised staging system is more relevant for the precise study of the developmental processes taking place during *A. filiformis* regeneration. It also allows more homogeneous sampling by identifying regenerating arms at specific stages, which are directly comparable, despite coming from animals with different regeneration rates.

An important observation arising from this study concerns the mode of brittle star arm regeneration. By looking at the development of skeletal elements in the 50 % DI arms we can observe the whole gradient of regenerative developmental stages from the oldest differentiated proximal metameric units to the newest un-differentiated distal metameric unit with skeletal primordia. However, the distalmost tip containing the terminal ossicle and podium is differentiated, and the growth zone adding new metameric units is located just proximally to it. This suggests that after a transient stage of formation of the regenerative bud, the brittle star regenerates its arm following a distalization-intercalation model similar to what has been described in sea star [25] and planarian regeneration [4]. According to this model regenerating organisms first form the distalmost part of the regenerate, which acts as a re-organization centre, and then add new structures by sequential intercalation of newly generated tissues between the distal part and the stump following a distal-proximal gradient. The following observations are consistent with this model of regeneration: 1) the small amount of EdU^+^ cells in the distalmost tip of late regenerates compared with the underlying growth zone (Fig. 8C) supports the differentiation status of the distalmost element; 2) the early appearance of skeletal elements in the distalmost tip, which suggests early differentiation of the terminal ossicle (stage 5, Fig. 4a); 3) the appearance of forming segments in an intermediate position between the terminal tip and the stump (Fig. 4). This is consistent with gene expression studies of transcription factors known to be involved in early specification of mesodermal cells (e.g., *alx1, ets1/2, gataC*) and reported to be expressed in the growth zone, but not in the distalmost tip [37]. A similar mode of regeneration has been recently reported for sea stars, in which the distalmost element is represented by the terminal tube foot and associated terminal ossicle and the growth zone is located just at the base of this structure [32]. Whether the terminal structures of both brittle stars and sea stars have ‘simply’ a protective function over the delicate growth zone or act as true signalling centres to the patterning of regenerating tissues remains to be elucidated.

Our observations of the formation of the skeleton during early regenerating stages suggest that cells undergo specification and differentiation events very early during the regeneration process. The spicule primordia observed at stage 3 resemble the granule-like skeletal rudiment of *A. filiformis* embryos [36], and both sea urchin embryos [11, 47] and juveniles [13] at the earliest step in the development of the skeleton, which then extend into tri-radiated and tetra-radiated spicules. The 50 % differentiated arm shows the developmental progress of the skeleton from single spicules up to the formation of complex mesh-like structures of the dermal plates (lateral, oral and aboral shields), spines and vertebrae. Notably, the vertebral spicules, which are internal skeletal elements, appear much later than those involved in the formation of the lateral shields and spines. As seen in SEM images, the complete vertebrae in adult non-regenerating arms of ophiuroids are composed of two conjoined ambulacral plates [28, 48, 49], which could explain why during regeneration the vertebrae appear to form by a fusion of spicule complexes from bilateral halves of each segment. The same SEM studies also show that they are clearly the most complex and dense skeletal elements in the ophiuroid arms [28, 48, 49]. Taken together, these data suggest molecular differences and possibly a separate developmental program might be involved in the formation of those internal-most skeletal structures compared to the sparser stereom constituting the lateral shields and spines. This is supported by differences in expression of *Afi-c-lectin*, present in all skeletal territories (Fig. 6), and *Afi-p19* and *Afi-p58b*, which are localized preferentially in the vertebrae in late regenerates (Additional file 1: Figure S5). Certainly different positional cues must be required for different skeletal elements. While it is conceivable that the epidermis acts as a signaling center for the underlying dermal layer of skeletogenic cells, as the ectoderm provides essential positional cues in the sea urchin embryos [50], this is unlikely to be the case for the skeletogenic cells forming the vertebrae. It would be interesting to test what potential signaling pathways might be involved in the formation of individual skeletal ossicles during brittle star arm regeneration. The VEGF and FGF signaling pathways have both been shown to play important roles in the development of embryonic skeleton in sea urchins [50–52], as well as in vertebrates [53, 54], such as directing skeletogenic mesoderm cell migration. The expression of the ligands and receptors of these pathways have additionally been characterized in brittle star embryos and sea star juveniles, and are consistent with the potential role of these pathway components in skeletogenesis also in other echinoderm phyla [55]. It would thus be compelling to investigate their role in the developing skeletal elements in the context of *A. filiformis* adult arm regeneration, as it could potentially reveal similar molecular components underlying skeletogenesis among deuterostomes despite the fact that skeleton of vertebrates and ambulacrarians is considered to have evolved independently.

Skeletal regeneration is observed in other deuterostome groups: for example in cirri regeneration of amphioxus [56], and in appendage regeneration of different vertebrates (reviewed in [6]). It has even been suggested that adult bone repair and regeneration may recapitulate embryonic bone development at a molecular level [57]. Although the skeleton of echinoderms is composed of calcium carbonate, instead of calcium phosphate, similarities of its ontogeny can be observed when compared to vertebrates. For example, in both groups of animals the trunk skeletal precursor cells are mesoderm-derived, motile mesenchymal cells [44, 47, 58] suggesting conserved features of skeletogenesis in deuterostomes. In sea urchins and brittle stars the larval skeleton is formed by a group of mesenchymal cells (skeletogenic mesoderm, for *A. filiformis* see Additional file 1: Figure S4B), which ingress into the blastocoel prior to gastrulation, then migrate and secrete the molecules (proteins, inorganic matrix, etc.), which form the biomineralized spicules [36, 44, 47]. In regenerating adult arms of *A. filiformis*, we observed that the first skeletal elements are deposited within the dermal layer of the early regenerate, coinciding with the appearance of a pool of mesenchymal cells localized in this domain (Figs. 1, 2 and 3). Cell tracing experiments would be required to conclude that these mesenchymal cells are directly responsible for the deposition of the biomineralized skeleton. On the other hand, the molecular signature of the mesenchymal cells in the dermis (determined by examining the expression of *Afi-c-lectin, Afi-p19* and *Afi-p58b)* provides additional evidence to support their role in skeleton development in light of the extensive knowledge of echinoderm skeletogenesis [11, 12, 14, 16, 25, 46]. All three genes are homologs of well-characterized biomineralization genes found in sea urchin larvae [45] and adults [17, 18] and expression of the two latter genes in the skeletogenic mesoderm of *A. filiformis* embryos was recently shown [36]. Their highly restricted expression patterns differ significantly from the expression of previously described mesodermal transcription factors such as *Afi-alx1, Afi-ets1/2* or *Afi-gataC* that, although also absent from the epidermis, occupy a broader domain extending from the dermal layer into the remaining sub-epidermal tissue layers [37]. Late in regeneration, the *Afi-c-lectin* expression pattern highly resembles the expression of *Afi-αcoll* [37], a homolog of the vertebrate *col2A1*, a widely conserved skeletogenic collagen gene [59, 60].

We show that the regeneration of the brittle star arm is associated with a high degree of cell proliferation. Importantly, we show that *Afi-c-lectin* expressing cells are not labeled with EdU at any time during regeneration; however, their number increases during regeneration, as does the number of skeletal structures. This suggests that there must be a constant supply of *Afi-c-lectin* expressing cells, likely constituting the skeletogenic cell population, since they themselves have no proliferative capacity. For example, a small pool of local progenitor cells that proliferate and give rise to daughter cells, which then molecularly and morphologically differentiate, could maintain the cell population. The newly formed cells could also be supplied from the adjacent coelomic epithelium, which shows high levels of cell proliferation (Fig. 7) and also expresses genes like *Afi-alx1* and *Afi-ets1/2* [37]. Alternatively, the radial water canal has also been implicated as a major source of cells in the regenerative bud [27] and, as mesenchymal cells, skeletogenic cells could migrate from there into their final dermal location. It is possible that epidermal signals could be involved in guiding the cells into the appropriate position and cause them to differentiate and stop proliferating, similar to what has been shown in the sea urchin embryo [50].

Many questions remain to be answered concerning the origin of the skeletogenic cells in the regenerating arm and the signaling cues required for the correct positioning and shaping of the spicules, their extension, and formation of highly complex and divergent skeletal elements. Our work provides the basis for the further study of this crucial developmental process.

## Conclusions

Here, we describe for the first time the developmental process of skeletal regeneration in a brittle star. Using different approaches we found that the cells expressing skeletal markers are localized in the dermal layer of the regenerating arm, where the deposition of the biomineralized skeletal spicules occurs during the regenerative process. During late regeneration stages these cells are arranged in a tightly controlled pattern that follows the shape of five skeletal elements of the adult arm, namely the oral, aboral and lateral shields, spines and vertebrae. Furthermore, no cell proliferation has been detected in the cell layer where the skeleton is synthesized. We thus conclude that the skeletogenic cells are likely to come from another source, which constantly supplies new cells to build the complex skeletal elements. Elucidating the cellular and molecular mechanisms of skeleton regeneration in brittle stars, including gene regulation involved in skeletogenic cell specification, and the differentiation, patterning, and origin of these cells in the regenerate, will greatly enhance our understanding of the mechanisms responsible for the extensive regenerative potential of echinoderms.

## Methods

### Animal handling, sample preparation and fixation

Adult *A. filiformis* were collected at the Sven Lovén Centre for Marine Sciences in Kristineberg, Sweden, and transported to London where they were kept in flow-through tanks with filtered artificial seawater (ASW; Instant Ocean, Aquarium Systems, 30‰ salinity) at 14 °C. After a period of acclimatization of at least one week the animals (disc sizes between 0.5 and 1 cm and arm lengths up to ten times the length of the body) were anaesthetized in 3.5 % MgCl_2_(6H_2_O) solution. The arms were then amputated (maximum two per animal) 1 cm from the disc and left to regenerate until they reached the desired stage (1–8 days for early stages and approximately 2 weeks for the 50 % DI stage). The regenerating arms were collected leaving 2–3 non-regenerating segments and then fixed in Bouin’s fixative at 4 °C for at least 2 weeks for histological sections or in 4 % PFA in 1x PBT (Phosphate buffer saline, 0.1 % Tween-20) overnight at 4 °C for *in situ* hybridization experiments. To show the individual adult skeletal ossicles the non-regenerating arms of the brittle star were amputated as described before and placed in 1 % bleach for approximately 20 min to degrade the tissue prior to imaging.

### Calcein staining

Live animals were incubated with calcein (Sigma) at a dilution of 1:50 of a stock solution (1.25 mg/ml) in artificial seawater to label the newly deposited calcium carbonate and thus visualize the biomineralized structures during the early stages of arm regeneration. Calcein was replenished every day together with adding fresh ASW throughout the duration of the observation period. The animals were washed in filtered seawater several times and anaesthetized in the magnesium chloride solution prior to imaging.

### Sectioning of paraffin-embedded tissue

PFA-fixed samples for thick sections were first de-calcified in 0.5 M EDTA for 1-3 days at 4 °C and then washed several times with PBT. The Bouin’s (VWR) fixed samples were washed several times in deionized water. Both types of samples were dehydrated in a series of increasing ethanol concentrated washes (30, 50, 70 %, 2× 100 %), washed twice (once at room temperature and once at 60 °C) with Histo-Clear (Fischer-Scientific) and then 3 times in paraffin wax at 60 °C (Thermo Scientific) before finally orientating them during the embedding. The wax was allowed to cool down and solidify overnight at room temperature. Samples were then mounted onto wooden blocks and sectioned at a thickness of 5–10 μm using a Leica RM2155 microtome, floated on slides with distilled water at 42°C and then left to dry overnight at 37 °C before further processing. The sections were de-waxed using Histo-Clear and processed for ISH or histology.

### Histology

Milligan’s trichrome technique was employed for the histological staining of Bouin’s fixed sections with modifications in the timing of staining of acid fuchsin (3 min) and fast green (8 min) [43]. All sections were de-waxed, rehydrated, stained and mounted with coverslip using Histomount (National Diagnostics) before imaging. Each stage.

### *In situ* hybridization

Probe preparation and whole mount *in situ* hybridization were performed as described before [22]. The *Afi-c-lectin* sequence can be found in GenBank under the accession number KT936152. At least three arms from different animals were used per experiment. Fluorescent *in situ* hybridization was performed on sections on microscopy slides using the protocol described in [36] with the modification of hybridization temperature (50°C). DAPI (Sigma) was added at a dilution of 1:5000 (of stock solution 5mg/ml) for 15 min and then washed out once with buffer just before microscopy.

### Cell proliferation assay

The cell proliferation assay was carried out using the Click-iT® EdU Alexa Fluor® 555HCS Assay (Life Technologies), which uses a modified thymidine analogue EdU that gets incorporated into newly-synthesized DNA and can be detected by fluorescent dyes using the highly selective copper-catalyzed azide-alkyne cycloaddition chemistry (click chemistry; [61]), without the need for antibody detection or harsh permeabilization treatments. Animals with non-regenerating arms and with arms at early regeneration stages were incubated in 5μM EdU in ASW for 2h, then the regenerating arms were amputated and fixed for 1h in 4 % PFA in PBT at room temperature. The arms were then washed with 1x PBT several times and permeabilized for 1h in 1x PBS with 0.1 % Triton X-100. After two more PBT washes 100μl of the reaction cocktail were added to the samples for 30 min (made according to manufacturer’s instructions and using kit reagents). The solution was then removed and the samples were washed for 30 min in Click-IT reaction rinse buffer. The buffer was then also removed followed by two PBT washes and finally DAPI was added as described above.

### Light and confocal microscopy

For differential interference contrast (DIC) images as well as epi-fluorescent images the Zeiss AxioImager M1 microscope was used together with a Zeiss AxioCamHRc camera. For confocal images of fluorescently labeled samples the Leica TCS SP2 confocal laser scanning microscope was used and the LAS-AF software implemented to capture the image stacks. Between 30 (for stage 2) and 109 (stage 3) confocal z-planes were taken at 1μm thickness for each confocal stack at early stages, and 90 z-planes at 1μm thickness were used for the 50 % regenerated arm samples. Images were processed using Fiji and Adobe Photoshop CS4.

## Additional files

Additional file 1: Additional figures and tables; Contains figures and tables supporting data in the main text. (PDF 151495 kb) Additional file 2:
**Movie S1**: Progression through a merged confocal z-stack of proximal segments of 95% regenerated arm showing EdU-labeled cells (red), *Afi-c-lectin* labeled cells (green) and DAPI-labeled nuclei (blue). Individual z-planes show no cells with both red EdU nucleus and green *Afi-c-lectin* labeled cytoplasm. (AVI 2760 kb) Additional file 3:
**Movie S2**: Progression through a merged confocal z-stack of distal tip of 95% regenerated arm showing EdU-labeled cells (red), *Afi-c-lectin* labeled cells (green) and DAPI-labeled nuclei (blue). Individual z-planes show no cells with both red EdU nucleus and green *Afi-c-lectin* labeled cytoplasm. (AVI 3205 kb)
