# Supplementary material for: Skeletal regeneration in the brittle star Amphiura filiformis
Source: Front Zool. 2016 Apr 22;13:18. doi: 10.1186/s12983-016-0149-x (PMC4841056; doi:10.1186/s12983-016-0149-x)
Supplement: Additional file 1: — Additional figures and tables; Contains figures and tables supporting data in the main text. (PDF 151495 kb) [file 12983_2016_149_MOESM1_ESM.pdf]

SUPPLEMENTARY FIGURES

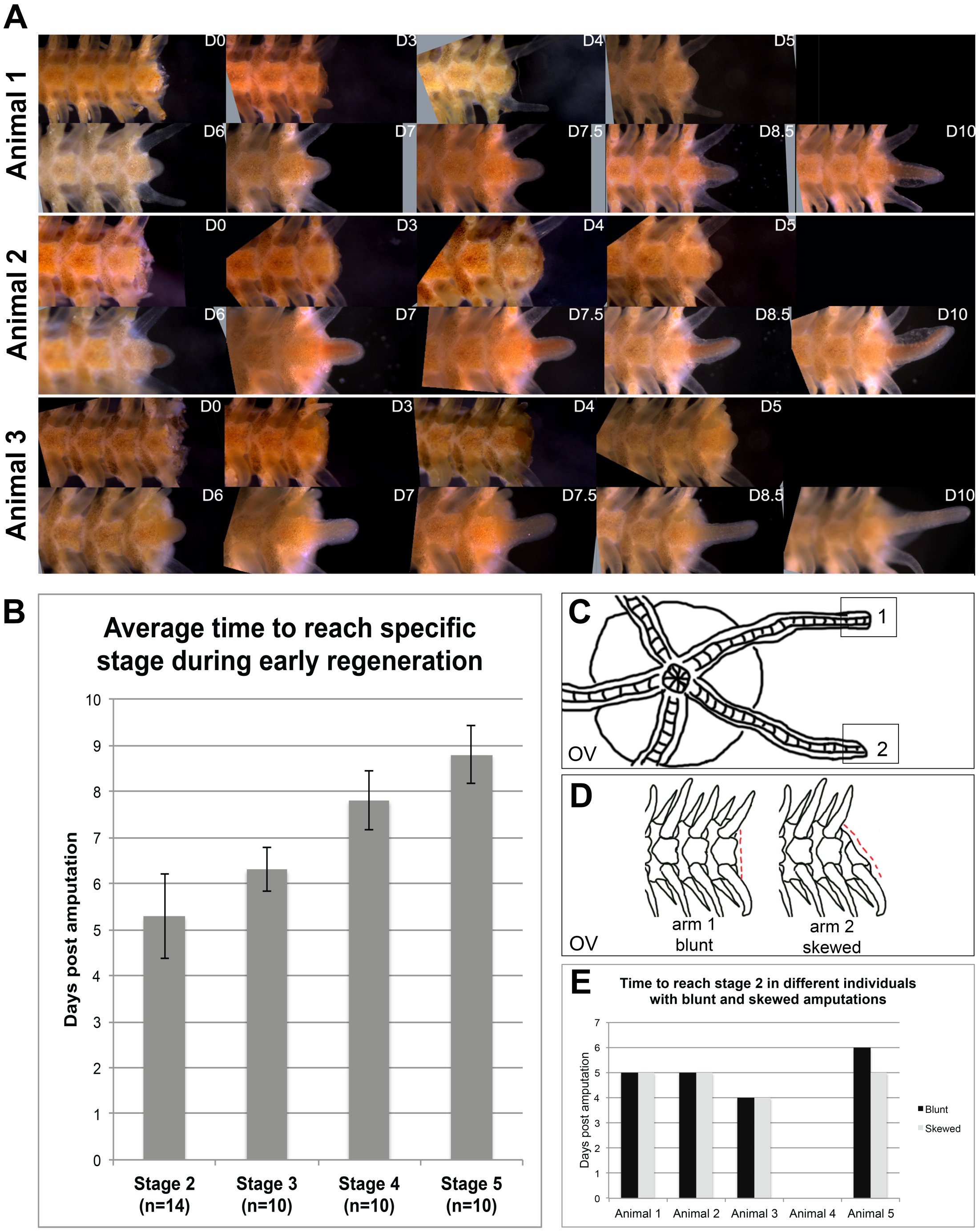

Figure S1: Staging of *A. filiformis* early arm regeneration based on morphological landmarks. A) Three individual brittle star arms showing individual variability in the regeneration rate. B) Graph showing averaged variability in time it takes for arms to reach stage 2, 3, 4 and 5 of early regeneration. Error bars show standard deviation between samples. C) A schematic diagram of the oral side of an adult *A. filiformis* with two arms amputated 1cm from the main body. Top arm has a blunt amputation and bottom arm has a skewed amputation plane. D) Schematic diagrams of blunt and skewed amputation planes of arms in C. E) Five individual animals of the same size were amputated 1cm from the main body disc and left to regenerate to stage 2. The graph shows individual animal variability in time to reach stage 2 but almost no difference between blunt and skewed amputated arms. Note animal 4 did not regenerate at all during this time and died shortly after the experiment. *D – days post amputation*, *OV – oral view*.

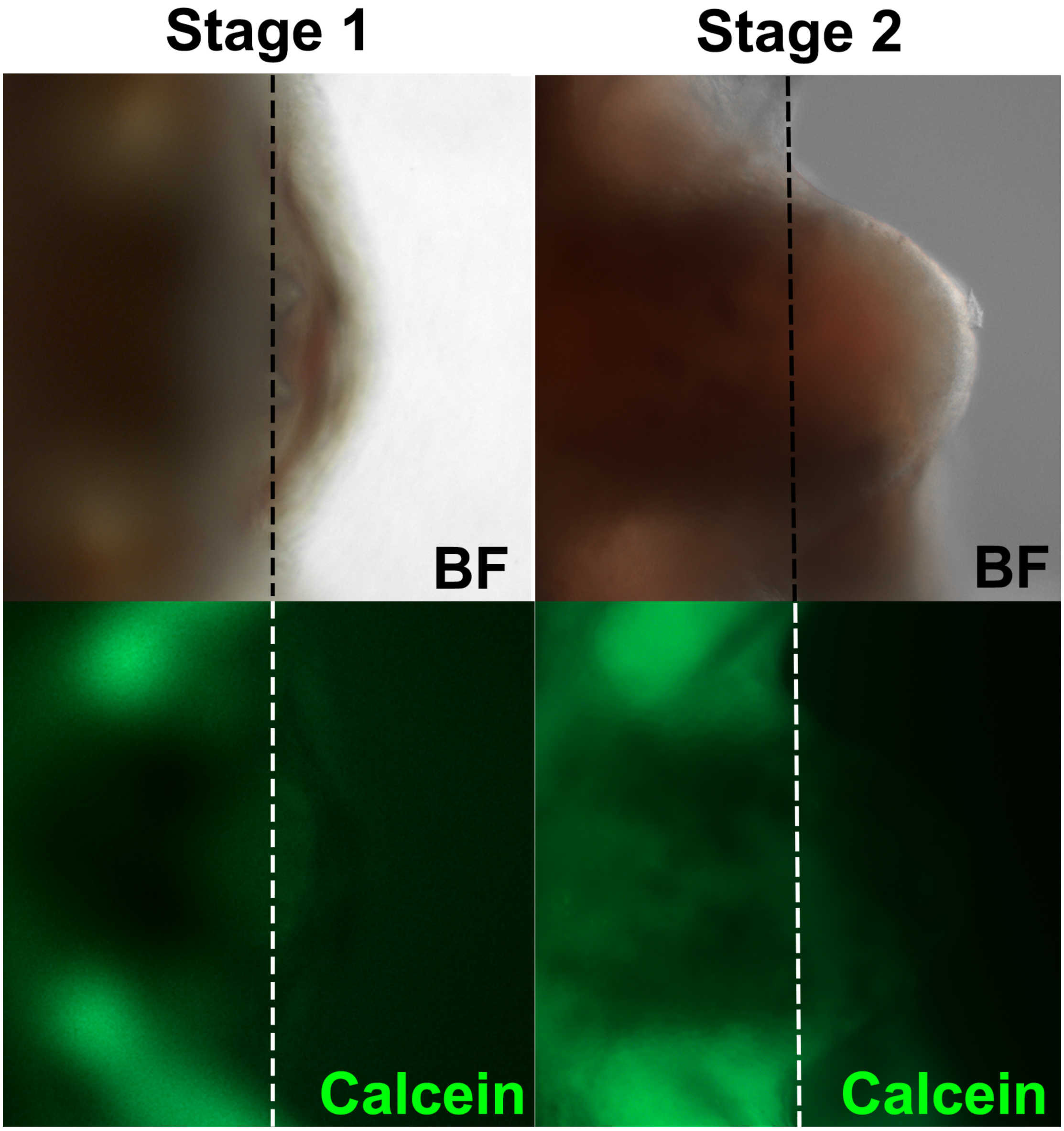

Figure S2: Calcein staining in early regeneration stages 1 and 2 showing that no skeletal elements are formed in the regenerate before stage 3. *BF – bright field*, *Dashed line – amputation plane*.

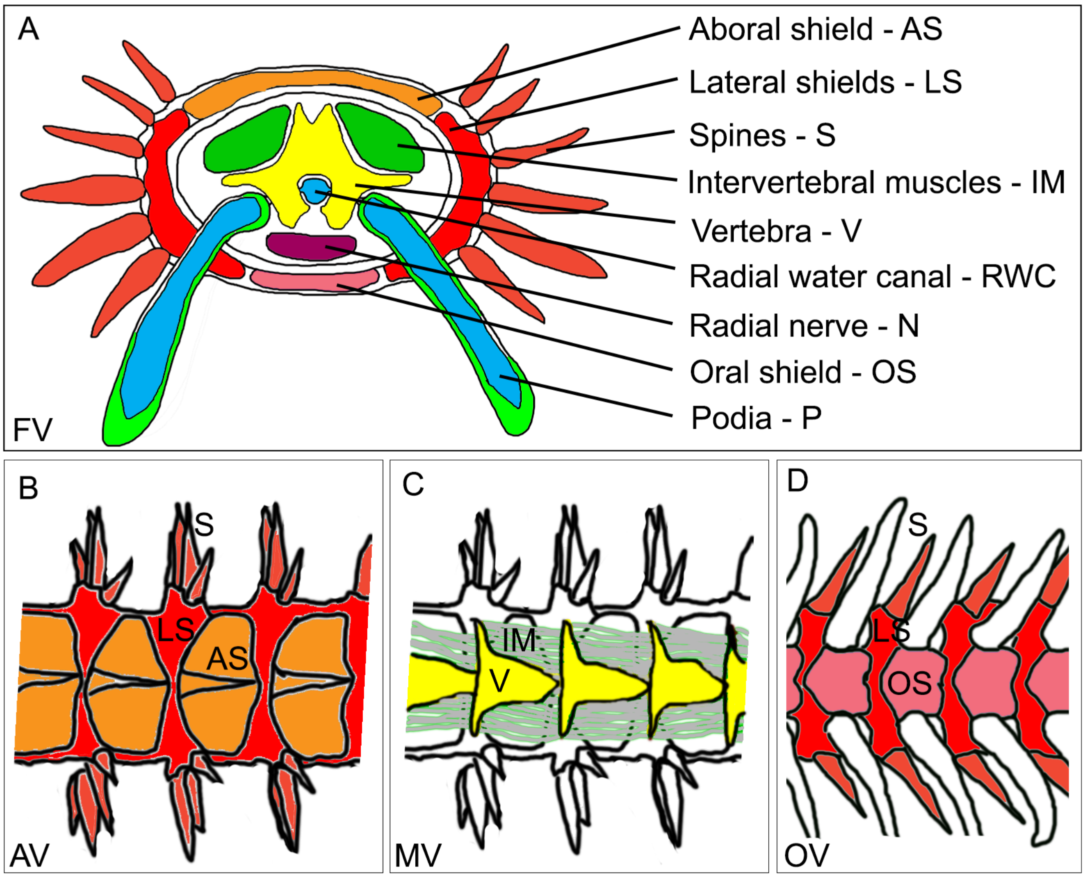

Figure S3: Schematic diagrams showing organization of adult non-regenerating *A. filiformis* arm. A) Frontal view showing the arrangement of skeletal elements in an individual segment of the adult brittle star arm and the position of internal structures. B) Aboral view of several segments of adult arm showing the position of the aboral and lateral shields and spines. C) Middle inner view of several adult arm segments showing the position of the vertebrae and the intervertebral muscles. D) Oral view of the adult arm showing the oral and lateral shields and spines. *AV – aboral view, FV – frontal view, MV – middle inner view, OV – oral view.*

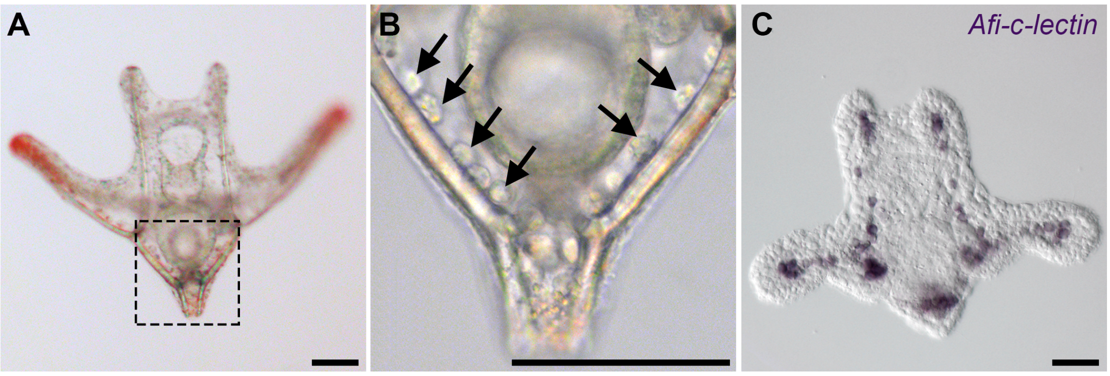

Figure S4: *A. filiformis* pluteus stage embryo showing the larval skeleton, associated skeletogenic cells and the expression of *Afi-c-lectin*. A) Live pluteus larva of *A. filiformis* with extended arms. The skeleton is visible as a birefringent structure providing the larva with its pluteus-type shape. B) Detail of A showing skeletogenic cells closely associated with the biomineralized skeletal rods in the apex of the pluteus. C) The pattern of *Afi-c-lectin* expression closely mirrors the organization of the elaborate larval skeleton. *Arrows – individual skeletogenic cells, Scale bars - 50µm.*

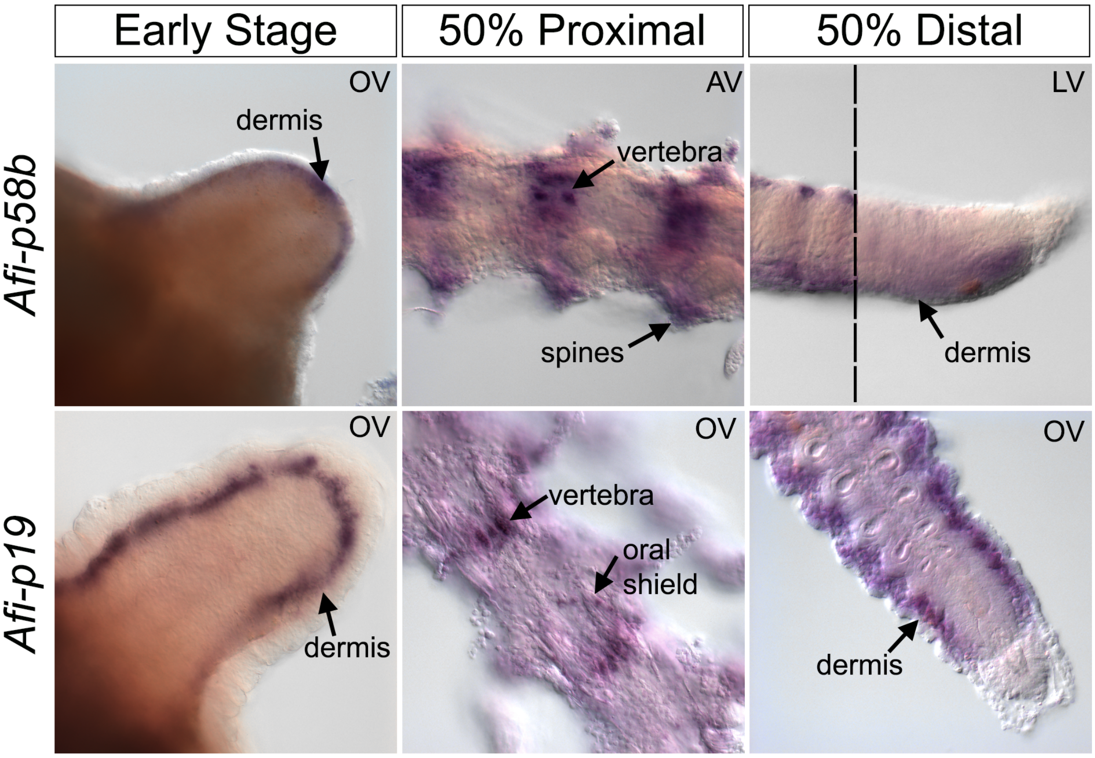

Figure S5: Spatial expression patterns of *Afi-p58b* and *Afi-p19* during early and late regeneration. Both *Afi-p58b* and *Afi-p19* are expressed specifically in the putative skeletogenic cells in the dermal layer of the regenerate both at the early stage of regeneration (stage 3-5) and in the distal area of the 50% regenerated arms. In proximal segments of the 50% DI arm *Afi-p58b* is expressed in the developing vertebrae and spines, *Afi-p19* is expressed in the developing vertebrae, oral and lateral shields. Dashed line indicates two different focal planes. *AV – aboral view, LV – lateral view, OV – oral view.*

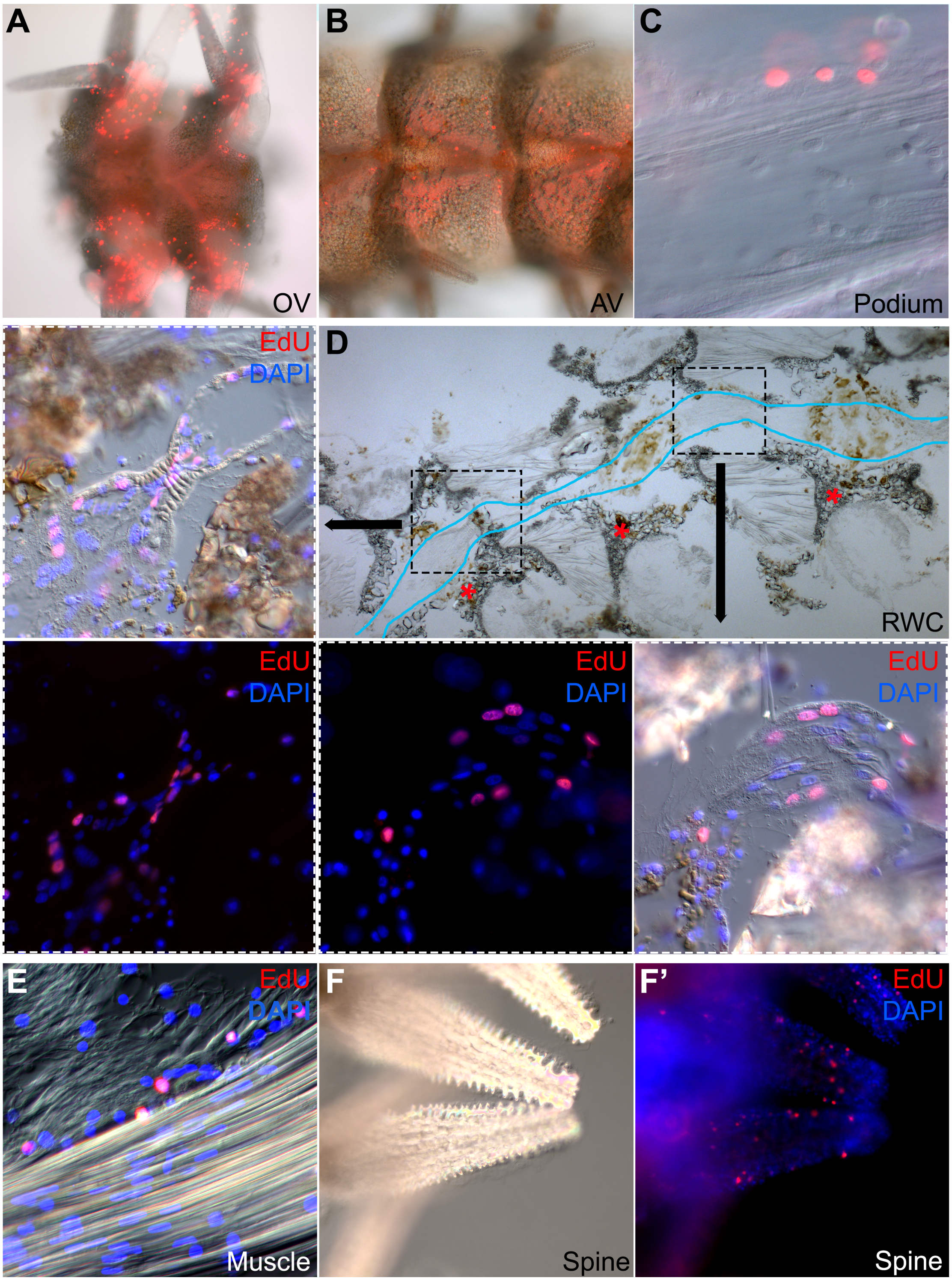

Figure S6: EdU labeling in the non-regenerating arm of *A. filiformis*. A) Proliferating cells are normally present in the non-regenerating arms around the oral shields, in the spines and in the podia. B) Proliferating cells are also present in non-regenerating arms in the epidermis covering the aboral shields. C) Detail of podium labeled with EdU shows that proliferating cells are localized predominantly in the epidermis of the podium. D) A frontal paraffin section through a non-regenerating arm showing the position of the radial water canal (blue) and vertebral remains (asterisk). Insets show EdU labeling of cells both inside the radial water canal and in its' epithelium. E) A frontal paraffin section of adult intervertebral muscles showing no red nuclei within the muscle cells but some labeled cells can be observed in close proximity of the muscle. F and F') Magnification of highly calcified adult spines showing several EdU-labeled cells. *AV – aboral view, OV – oral view, RWC – radial water canal.*

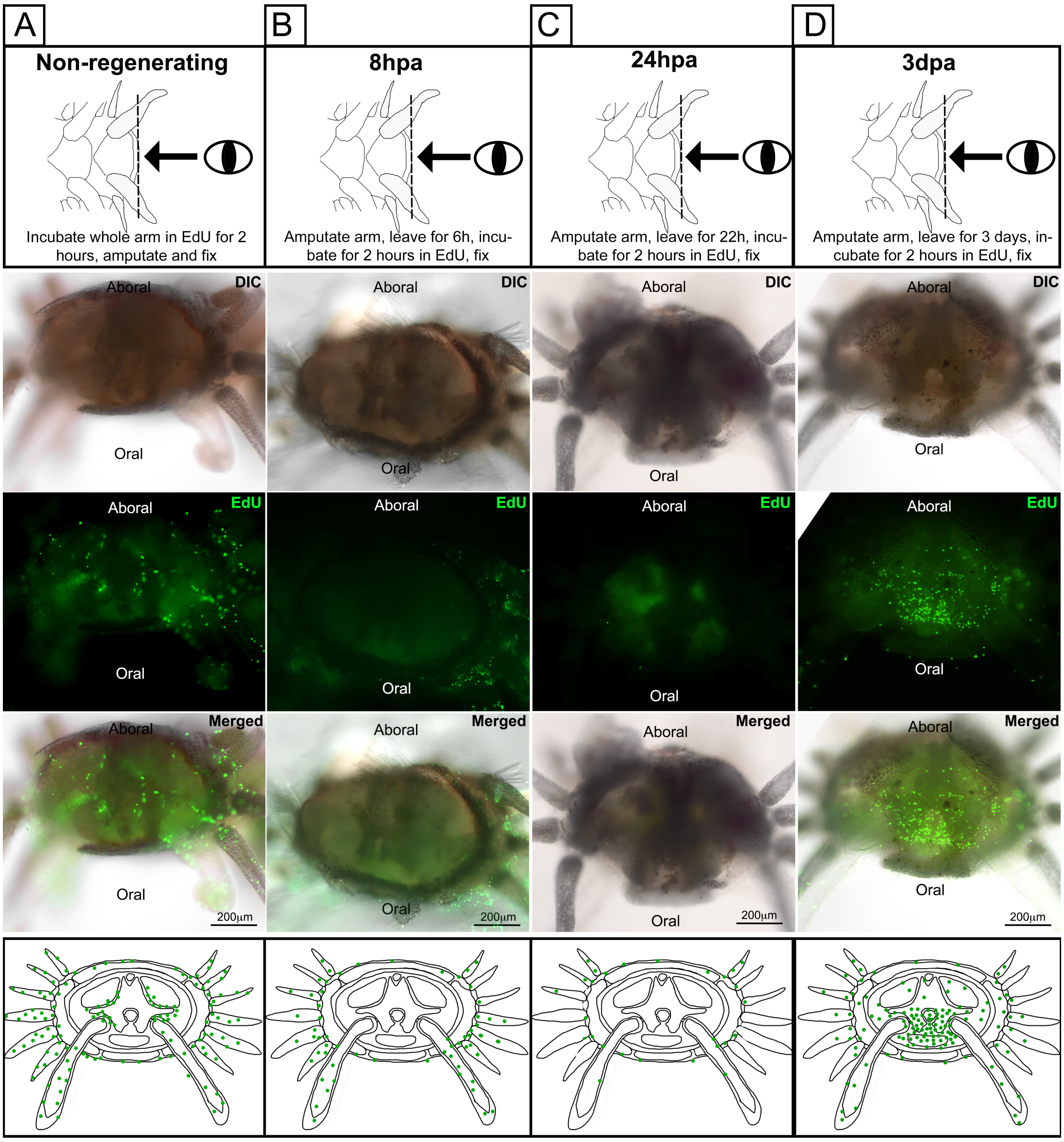

Figure S7: EdU-labeling showing proliferation in non-regenerating arms (n=5) and at stage 1 of regeneration (n=3) at different time points post amputation. Top panels A-D) Schematic diagrams showing the labeling method and imaging view. Middle panels A-D) Bight field, fluorescent, and merged images of EdU-labeled arms showing the frontal view of the amputation and wound healing site. Bottom panels A-D) schematic diagram of frontal view of arm stump summarizing cell proliferation at different time points. A) EdU labeling pulse (green) in non-regenerating arms amputated to reveal frontal view of internal structures. Cell proliferation is observed in the epidermis, spines, and podia and in cells surrounding the vertebra. B) EdU pulse in stage 1 arms at 8 hours pa shows and almost complete shutdown of cell proliferation in the early phase of wound healing. Some proliferating cells are still observed in the stump in the epidermis and spines. C) EdU pulse in stage 1 arm at 24hpa shows cell proliferation is still absent during the epithelialization phase. D) At end of stage 1, at 3 days pa, EdU labeled cells can be detected at the amputation site and an accumulation of proliferating cells can be observed in the central-oral position corresponding to the site of the future appearance of the regenerative bud.

SUPPLEMENTARY TABLES

Gene name	Sequence source	Accession number	Blast	Closest hit	Score (bits)	E Value
Afi-c-lectin	*Amphiura filiformis*	KT936152 (NCBI)	Echinobase	Sp-C-lectin (SPU_007882)	98	8e-21
			NCBI	Ow-Sm22 (AJT58576.1)	145	1e-40
Sp-c-lectin	*Strongylocentrotus purpuratus*	SPU_007882 (Echinobase)	Afi transcriptome	Afi-C-lectin (KT936152)	99.8	6e-25
Ow-sm22	*Ophiocoma wendtii*	AJT58576.1 (NCBI)	Afi transcriptome	Afi-C-lectin (KT936152)	154	3e-47

Table S1: BLAST results for *Afi-c-lectin* showing closest sequence match to *S. purpuratus c-lectin* and *O. wendtii sm22* genes. *Afi – Amphiura filiformis, Sp – Strongylocentrotus purpuratus, Ow – Ophiocoma wendtii.*

**Arms scored**	**Stage**	**Segments**	**Separate z-planes scored**	**Green signal** (*C-lectin* in cytoplasm)	**Red signal** (EdU in nucleus)	**Potential overlap***
**Arm 1**	95% proximal	2	60	214	287	2
**Arm 2**	50% proximal	2	60	71	381	1

* Potential overlap refers to either yellow signal due to overlap between green cytoplasmic staining of *Afi-c-lectin* in one cell and red nuclear EdU labeling of another cell or a true cell overlap where a cell has both a red nucleus and surrounding green cytoplasm. See figure 8 for image representation.

Table S2: Scoring of EdU and *Afi-c-lectin* double-labeled arms.
